# Pharmacogenetics of CYP3A5 on Carbamazepine pharmacokinetics in epileptic patients developing toxicity

**DOI:** 10.1186/1471-2164-15-S2-P2

**Published:** 2014-04-02

**Authors:** Mubarak Al-Gahtany, Gauthaman Karunakaran, Murali Munisamy

**Affiliations:** 1 Faculty of Neuro Surgery, King Khalid University, Abha, KSA; 2 Department of Pharmacology, College of Pharmacy, King Khalid University, Abha, KSA

## Background

The genetically polymorphic cytochrome P450 enzymes are involved in the metabolism and elimination of a number of widely used drugs. CYP3A5 exhibits remarkable inter-individual differences in the pharmacokinetics of Carbamazepine [1]. The present study was undertaken to investigate the effects of CYP3A5 on the pharmacokinetics of antiepileptic drug Carbamazepine in the epileptic patients showing toxicity.

## Materials and methods

30 epileptic individuals who had developed toxicity to carbamazepine and 30 control epileptic subjects who had not developed toxicity to carbamazepine were genotyped for CYP3A5 polymorphisms by polymerase chain reaction-restriction fragment length polymorphisms (PCR-RFLP Method). Carbamazepine plasma levels were analyzed by reversed phase HPLC method and pharmacokinetic parameters such as area under the concentration curve (AUC), maximum concentration (Cmax), time to Cmax (tmax) and half-life (t1/2) were estimated by non-compartmental analysis using PK SOLUTIONS® software.

## Results

A significant correlation was observed in the frequency of homozygous CYP3A5 mutant allele (P <0.01) among the carbamazepine toxicity and controls. The pharmacokinetics parameters of carbamazepine in homozygous mutant group showed longer half-life (t ½ = 17 hrs) and less clearance rate (CL =1.5 L/hr) when compared to wild type group ( t ½ =12.8 hrs, CL = 2.9 L/hr).

## Conclusions

Our findings suggest that the CYP3A5 Genetic Polymorphisms plays a significant role in the steady state concentrations of carbamazepine and thereby having impact on toxicity in epileptic patients.
